# Pharmacodynamics, toxicology and toxicokinetics of ropivacaine oil delivery depot

**DOI:** 10.1186/s12871-022-01653-1

**Published:** 2022-04-21

**Authors:** Wu-dang Lu, Min-quan Hui, Jing-liang Gu, Li Liu, Man-li Wu, Yi Yang, Yong-xiao Cao

**Affiliations:** 1 Department of Pharmacology, School of Basic Medical Sciences, Xi’an Jiaotong University Health Science Center, 76 Yanta West Road, Xi`an, 710061 Shaanxi China; 2 Xi’an Libang Pharmaceutical Co., Ltd., Xi’an, 710061 Shaanxi China; 3 Joinn Laboratories Co., Ltd., Suzhou, 215421 Jiangsu China

**Keywords:** Ropivacaine oil delivery depot, Rabbit, Pharmacodynamics, Rat, Toxicology, Toxicokinetics

## Abstract

**Background:**

Ropivacaine oil delivery depot (RODD) can be used to treat postoperative incision pain. The aim was to study pharmacodynamics, toxicity and toxicokinetics of RODD.

**Methods:**

The base research of RODD were conducted. Thirty rabbits were randomly divided into saline, solvent, ropivacaine aqueous injection (RAI) 0.9 mg, RODD 0.9 mg and RODD 3 mg groups. The sciatic nerve of rabbits were isolated, dripped with RODD and the effect of nerve block were observed. In toxicity study, the rats were divided into saline, solvent and RODD 75, 150 and 300 mg/kg groups, 30 rats per group. In toxicokinetics, rats were divided into RODD 75, 150 and 300 mg/kg groups, 18 rats per group. The rats were subcutaneously injected drugs.

**Results:**

The analgesic duration of RODD 3 mg and RAI 0.9 mg blocking ischiadic nerve lasted about 20 h and 2 h, respectively, and their blocking intensity was similar. The rats in RODD 75 mg/kg did not show any toxicity. Compared with saline group, in RODD 150 mg/kg group neutrophils and mononuclear cells increased, lymphocytes decreased and albumin decreased(*P* < 0.05), and pathological examination showed some abnormals. In RODD 300 mg/kg group, 10 rats died and showed some abnormalities in central nerve system, hematologic indexes, part of biochemical indexes, and the weights of spleen, liver, and thymus. However, these abnormal was largely recovered on 14 days after the dosing. The results of toxicokinetics of RODD 75 mg/kg group showed that the C_max_ was 1.24 ± 0.59 µg/mL and the AUC_(0-24 h)_ was 11.65 ± 1.58 h·µg/mL.

**Conclusions:**

Subcutaneous injection RODD releases ropivacaine slowly, and shows a stable and longer analgesic effect with a large safety range.

## Background

Postoperative pain is an acute, high-intensity pain with duration of 24⁓72 h. Mechanism research showed that chemicals or cytokines, including bradykinin, prostaglandins, serotonin, histamine, acetylcholine, etc. were released at the tissue injury sites after operation [1, 2]. These factors increases peripheral nociceptors to generate nerve impulses which enteres the central nervous system, and ultimately produces pain response. Fiber A conducts fast pain impulse, and fiber C mainly conducts chronic ache, thermal pain and postoperative pain impulse [3–5].

Ropivacaine, an S-enantiomer amide type of local anesthetic with a high pKa, showing low fat solubility and long action duration, effectively blocks fibers A and C. At low concentrations, ropivacaine has a better block effect on fiber C than on fiber A, which meets the clinical requirements for an analgesic [6, 7]. Sciatic nerve is blocked with 0.75% ropivacaine during operations, which reduces the dosage for postoperative analgesic drug. In addition, ropivacaine hydrochloride injection is used in brachial plexus block surgeries and the onset time is 10⁓45 min and the duration of the sensory block is 3.7⁓8.7 h [8, 9]. Sensory nerve block duration of ropivacaine aqueous injection (RAI) is too short to meet clinical requirements, so we developed ropivacaine oil delivery depot (RODD) in which ropivacaine is active ingredient, benzyl alcohol as the solvent and both benzyl benzoate and soybean oil as the dispersants. Our early studies indicated that RODD is retained for longer time than RAI as subcutaneous injection in dogs and pigs [10, 11]. Based on these results, we studied its pharmacodynamics, the acute toxicology and toxicokinetics of the subcutaneous injection of RODD in Xi’an and Suzhou, China.

## Methods

### Reagents and chemicals

Benzyl alcohol and benzyl benzoate were purchased from Merck Chemical Co., Ltd., Germany. Soybean oil was produced in Tieling Pharmaceutical Co., Ltd., China. Ropivacaine aqueous injection (RAI, 9 mg/mL) was obtained from Libang Pharmaceutical Co., Ltd., China. The ropivacaine reference was obtained from USP; and bupivacaine hydrochloride was obtained from the National Institutes for Food and Drug Control, China.

### Preparation of RODD and solvent

Ropivacaine 36.5 g was added to benzyl alcohol 100.8 g and benzyl benzoate 405.5 g, then heated, and stirred. After the ropivacaine was dissolved, soybean oil 651.3 g were added to the ropivacaine solution. Then, the mixture was stirred until the solution clear. After sterile filtration with filter, the bulk solution of RODD 30 mg/mL was obtained. The RODD was diluted with solvent to 9 mg/mL. The solvent was prepared with the same procedure without ropivacaine.

### Animals

Thirty New Zealand rabbits, male, 2.5 ± 0.5 kg, was provided by Slakey Experimental Animal Center, Shanghai, China (SCXK (Hu)0.2007–0005). Specific-pathogen-free (SPF) Sprague Dawley (SD) rats, 6⁓8 weeks and 180⁓220 g, were purchased from Beijing Weitong Lihua Experimental Animal Technology Co., Ltd., China (No.11400700175820). The animal were allowed to freely consume water. The houses were well ventilated, the temperature was 20 °C⁓26 °C, and the humidity was 40%⁓70%. All animals were conducted in compliance with the guidelines of ethical animal research. Procedures used in this study were approved by the Institutional Animal Care and Use Committee at Joinn Laboratories (No.ACU13-722). Animals were euthanized by intravenous injection of sodium pentobarbital after the study.

### Pharmacodynamics

#### Rabbit’s surgery

**Fig. 1:**
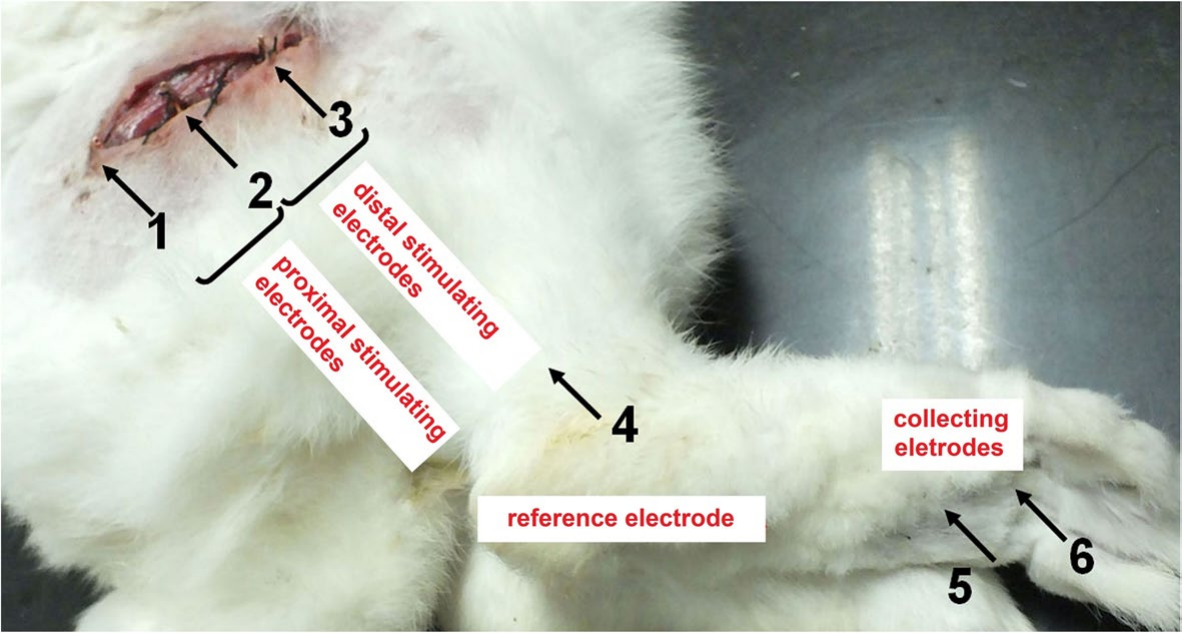
Schematic diagram of rabbit sciatic nerve surgery. Needle No.1 and 2 formed the proximal stimulating electrode of heart while the needles 2 and 3 formed the distal stimulation electrode of heart. No. 4 needle as a reference electrode, was inserted into the ankle. No. 5 needle inserted into the sole, and No. 6 needle inserted into the muscle between the first and second toes were composed a receiving electrode

Rabbits were anesthetized with 5% isoflurane at beginning and 2% isoflurane in the process of the surgery. A 5 cm incision was cut at the right hind leg to expose the sciatic nerve. As shown in Fig. 1, three acupuncture needles marked as No 1, 2 and 3, as stimulating electrodes, were respectively bent, hooked to the sciatic nerve, and fixed on muscle by sutures. Needle 1 and 2 formed the proximal stimulating electrode of heart, while the needles 2 and 3 formed the distal stimulation electrode of heart. The distance between the needles around was 1 cm. After the surgery, ampicillin 50 mg was injected intramuscularly to prevent infection [12]. The No. 4 needle, a reference electrode, was inserted into the ankle. No. 5 and No. 6 needles, as receiving electrodes, were inserted into the sole and the muscle between toes, respectively.

#### Grouping and measurement

Rabbits were randomly divided into 5 groups with 6 in each group as follows: saline (model) group; solvent group, RAI 0.9 mg group; RODD 0.9 mg and 3 mg groups. The exposed sciatic nerve on needles 2 and 3 were dropped 0.1 mL of drugs, respectively. The animal hip was raised to keep infiltration status for 10 min, then the wound was sutured. Voltage stimulus in the electrode 2 and 3 was applied until waveform became stable. The stimulate voltage, the time and peak of electrical signal in the electrode 5 and 6 were recorded. The nerve conduction velocity were calculated at 0, 1, 2, 3, 4, 5, 30 min; 1, 2, 4, 6, 8, 10, 12, 16, 20, 24 h after administrations. Nerve conduction velocity (m/s) = distance between proximal electrode of heart and telecentric electrode/ (conduction time between proximal and distal electrodes).

### Toxicology

#### Grouping and administration

In toxicology experiment 150 rats were divided into 5 groups with 30 rats per group: saline group, solvent group and RODD 75, 150 and 300 mg/kg groups, half male and half female. After fasting 12 h, the rats were subcutaneously injected with RODD (30 mg/mL) at multiple points, and the volume was not more than 1 mL per point. Rats in saline and solvent groups were given 10 mL/kg of saline or solvent, respectively.

#### General observation

Daily observations were performed to evaluate the external signs and behavioral activities of the rats, including mental condition, hair color and oral, nasal, eye, ear and external genital secretion. The analysis of toxicity included the evaluation of the central and motor nerve systems (behavior, movement, response to stimulation, neural reflex and muscular tension), vegetative nervous system (pupil and secretion), respiratory system (nasal and respiratory rate), urogenital system (labia, mammary gland and perineum), skin and hair (color and integrity), eye (eyelid and eyeball), ear (auditory meatus), etc.

#### Weight and food consumption

The rats were raised in individual cages and their body weights were measured before the experiment and on days 1, 4, 7, 11 and 14 of the experiment. Additionally, the food consumption of the rats within the first 24 h of the experiment were measured by the weight loss method.

#### Hematology

After the rats were anesthetized, blood was gathered from the abdominal aorta and the plasma was separated. Blood cell characteristics and blood biochemical parameters, including alanine amino transferase, albumin, albumin/globulin ratio, total protein, triglyceride and so on, were evaluated. Coagulation function were analyzed. Electrolytes such as total calcium, inorganic phosphorus, sodium, potassium ion concentration and chloride ion concentration were detected by an easylyte electrolyte analyzer.

#### Anatomy and pathological examination

The rats were euthanized on days 3 and 14. The body surface, orifice, cranial cavity, thorax, abdominal cavity, pelvic cavity and their contents were observed. The organs were separated and fixed in 10% neutral formalin solution, and the testicles were preserved with Davidson’s solution. The body and visceral organs were weighed, and visceral indexes were calculated, including the brain, thymus, thyroid gland, parathyroid gland, hepatic, kidney, adrenal gland, spleen, lung, heart, prostate, testis, epididymis, uterus, ovary, etc. The organs of the rats were treated with routine histological operations, including paraffin embedding, sectioning, preparation, HE staining, etc., and examined under a microscope.

### Toxicokinetics

#### Detection of blood ropivacaine concentration

The chromatographic conditions and methods of LC–MS/MS in the study were from the previous study [10] as follows: chromatographic column, Ultimate XB-C_18_ (4.6 × 150 mm, 5 µm); mobile phase, aqueous solution of 5 mM ammonium acetate:acetoni-trile (5:95 (v/v)); flow rate, 1.0 mL/min; injection volume, 10 µL; and run time, 4 min. The electrospray ionization source was an API 4000 Qtrap, ion trap mass spectrometry was used as the detector and a positive ion detection method was applied. The capillary voltage was 0.5 kV, the ion source temperature was 120 °C, the atomization temperature was 350 °C, the conical hole gas velocity was 150 L/h, and the atomized gas velocity was 650 L/h. Methodological validation included specificity, accuracy, limit of quantitation, precision, matrix effects and recovery, etc.

#### Grouping and testing

Fifty four rats were divided into RODD 75, 150 and 300 mg/kg groups with 18 rats per group, half male and half female. After fasting 12 h, RODD was injected subcutaneously on the back neck of rats at multiple points, and the volume was not more than 1 mL per point. Blood (0.5 mL) was extracted from the orbital veins at 0, 0.25, 0.5, 1, 2, 4, 8, 24, 48 and 72 h after injection. Plasma (50 µL) was added into 950 µL of biological matrix (containing 30 ng/mL internal standard). The protein in plasma was precipitated with methanol. Then, the samples were vortexed for approximately 1 min and centrifuged at 12,000 RPM for 10 min, the supernatant (20 µL) was accurately transferred and diluted with 380 µL of water. Finally, the solution was vortexed and analyzed by LC–MS/MS. The toxicokinetic parameters, including AUC, C_max_, T_max_, etc., were calculated in WinNonlin (V6.2) software.

### Statistical analysis

Data are presented as mean ± standard deviation (SD). The pharmacodynamics data were analyzed by one-way ANOVA. The toxicological data were tested for homogeneity of variance by Levene’s test and when the variance was homogeneous, one-way ANOVA was performed. If Levene’s test showed heterogeneity of variance, the Kruskal–Wallis nonparametric test was performed. The part of statistical analyses were conducted using GraphPad prism 6.0 (GraphPad Software, La Jolla, CA, USA). *P* < 0.05 was considered significant.

## Results

### Pharmacodynamics

#### RODD blocked sciatic nerve conduction

**Fig. 2:**
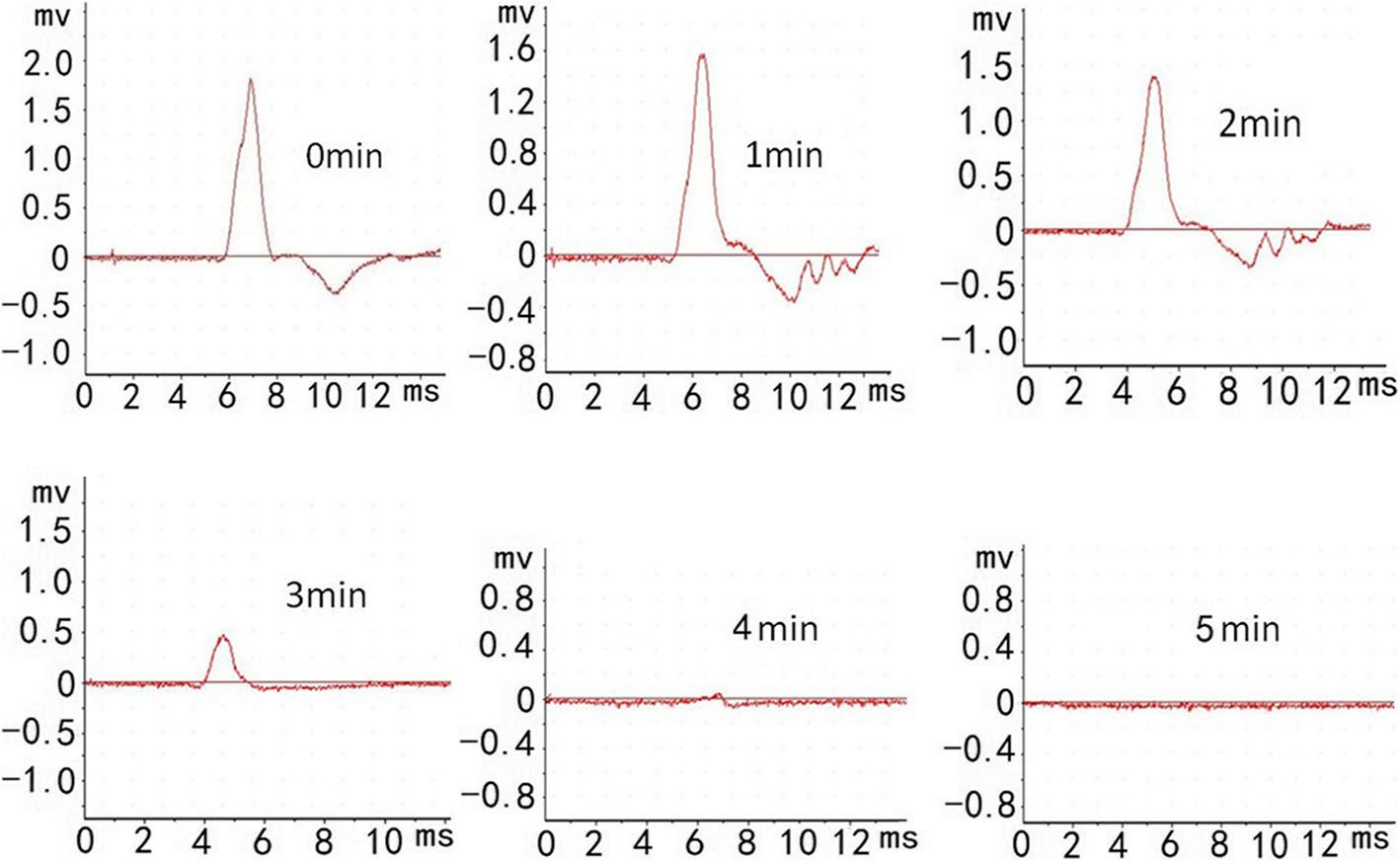
Rabbit sciatic nerve block electrogram in electrophysiograph. After administrated ropivacaine aqueous injection RAI 0.9 mg, nerve impulse conduction were obviously inhibited in 3 min and completely blocked in 5 min

**Fig. 3:**
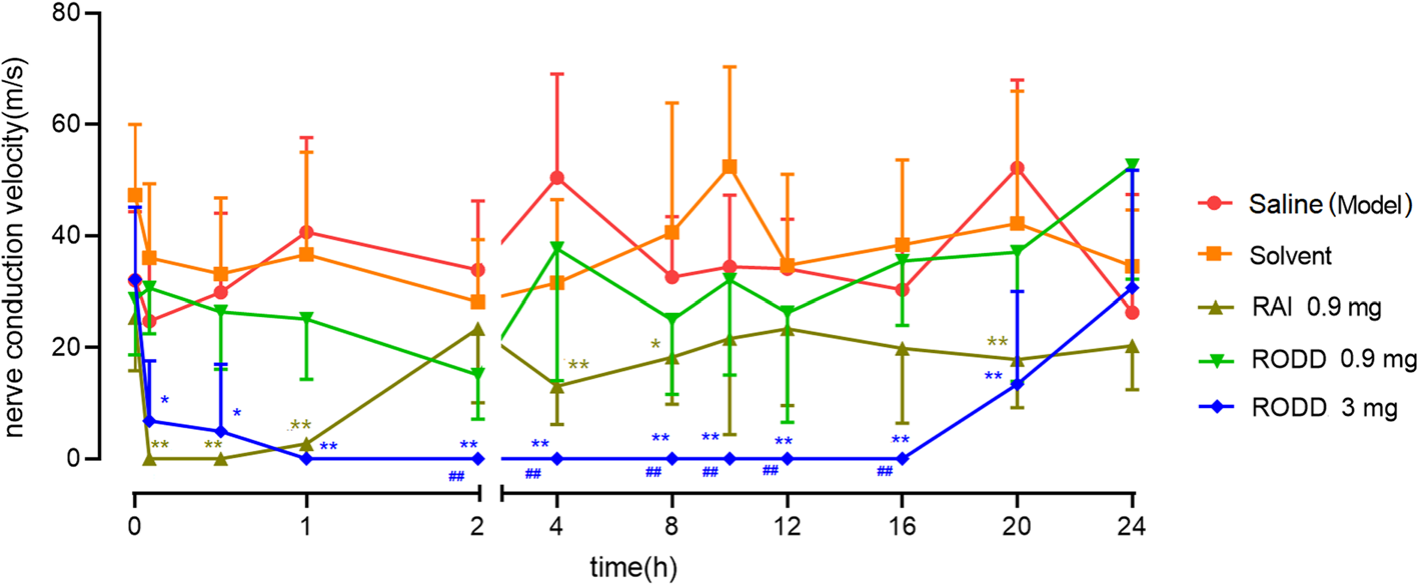
Blocking effects of RODD on nerve conduction. *n* = 6 (male). ^*^P < 0.05, ^**^P < 0.01 vs saline group, ^#^*P* < 0.05, ^##^*P* < 0.01 vs RAI 0.9 mg group. The nerve block effect of RAI 0.9 mg and RODD 3 mg was significant in 1 h after administration. After 2⁓3 h, the nerve block of RAI group gradually disappeared. RODD 3 mg inhibited nerve conduction for about 20 h, and the inhibition gradually disappeared. RODD 0.9 mg and solvent did no inhibit nerve conduction

Figure 2 showed that RAI 0.9 mg could blocked rabbit nerve conduction in 5 min. As shown in Fig. 3, the nerve block gradually disappeared about 2 h after administration. RODD 0.9 mg group showed a tendency blocking nerve conduction, but the difference was not significant(*P* > 0.05). RODD 3 mg could almost completely blocked the nerve conduction in 5 min. The nerve conduction completely disappeared in 1⁓16 h, and recovered in 24 h. The results showed that compared with normal saline group or RAI group, RODD 3 mg had a significant difference in the range of 1⁓20 h or 2⁓16 h (*P* < 0.01), respectively. Therefore, RODD 3 mg could produce an intense and long nerve conduction block.

### Toxicology

#### General observation

The rats in solvent group were characterized by prostration, decreased activity and lethargy on the 1st to 3rd days after administration. One male rat suffered incrustation at the injection site on day 14. In RODD 75 mg/kg group, one female rat had festered skin and incrustation at the injection site during the 8th to 14th day and other rats had no any unnormal behavior. The activity of two-thirds of rats in RODD 150 mg/kg group decreased 10 min after administration and recovered approximately 2 h later. In addition, one male rat had incrustation at the injection site on the 14th day. In RODD 300 mg/kg group, one male rat and nine female rats died 24 h after administration. Other rats showed polypnea, excitement/dysphoria, arched back, prostration, tremors, limb weakness, shaggy and lethargy within the first day after administration and 2 male rats had incrustation at the injection site on the 14th day.

#### Body weight and food consumption

**Fig. 4:**
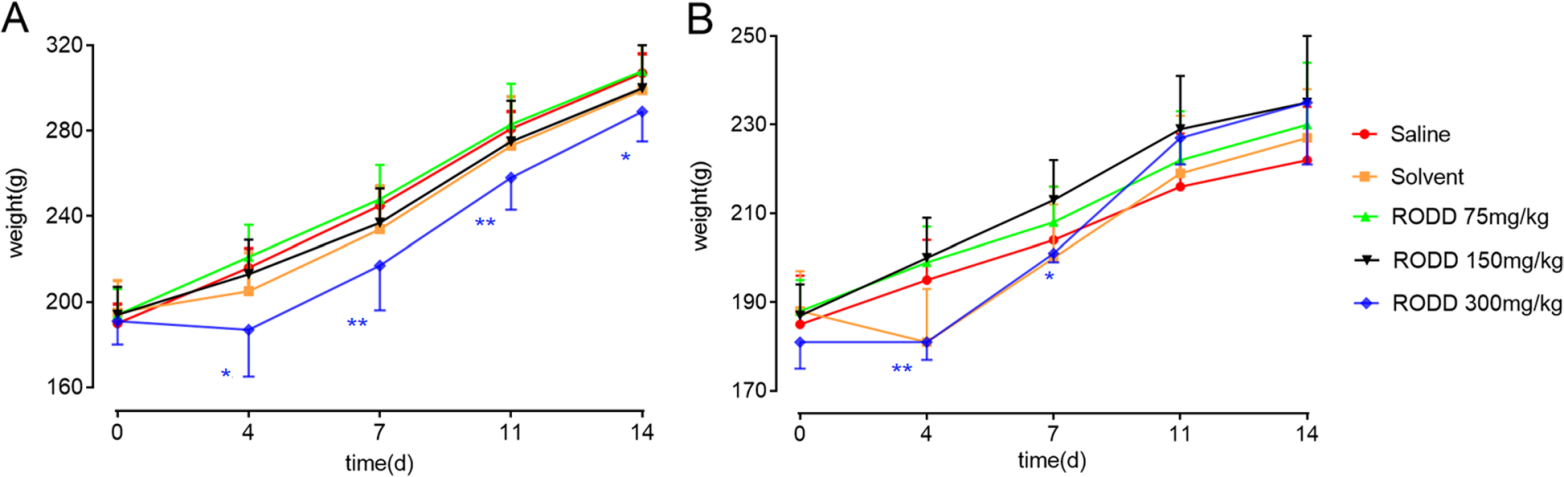
Weight-time curve after the subcutaneous injection of the RODD in rats. *n* = 30 (half male and half female). ^*^P < 0.05, ^**^P < 0.01 vs saline group. **A** represented male rats and **B** represented females

Compared with saline group, the male rats in solvent and RODD 300 mg/kg group and the female rats in solvent group had decreased food intake on the 1st to 4th days (19.8 ± 0.7 g, 15.9 ± 1.8 g, 12.2 ± 0.6 g vs 26.3 ± 0.3 g, *P* < 0.01). The Body weights of female rats in solvent group decreased compared with those in saline group on the 4th day (181 ± 12 g vs 195 ± 9 g, *P* < 0.05). Body weights of male rats in RODD 300 mg/kg group decreased on the 4th, 7th, 11th and 14th days (187 ± 22 g vs 216 ± 9 g, 217 ± 21 g vs 245 ± 9 g, 258 ± 15 g vs 281 ± 8 g, 289 ± 14 g vs 307 ± 9 g, *P* < 0.05, Fig. 4). Additionally, weight loss may be associated with reduced food consumption.

#### Blood cells

Compared with saline group, rats in solvent group, female rats in RODD groups and male rats in RODD 150 and 300 mg/kg groups presented increased neutrophils and mononuclear cells (*P* < 0.05) and decreased lymphocytes on 3rd day (Table 1). On the 14th day, males in RODD groups exhibited decreased lymphocytes with increased neutrophils and mononuclear cells (*P* < 0.05), however, these changes had no toxicological significance. Other indicators were almost restored to normal in rats.

**Table 1:** Effects of the subcutaneous injection of RODD on major blood cells in rats

Dose (mg/kg)	Sex	Neutrophils(× 10^9^/L)	Lymphocyte(× 10^9^/L)	Mononuclear(× 10^9^/L)
Saline	*M*	1.02 ± 0.34	9.01 ± 1.90	0.14 ± 0.02
	*F*	0.47 ± 0.12	8.99 ± 2.62	0.12 ± 0.04
Solvent	*M*	1.88 ± 0.45	9.32 ± 2.43	0.26 ± 0.08
	*F*	0.65 ± 0.22	8.10 ± 1.86	0.20 ± 0.08
RODD				
75 mg/kg	*M*	1.54 ± 1.51	11.25 ± 1.72	0.25 ± 0.16
	*F*	0.91 ± 0.19	8.55 ± 1.54	0.19 ± 0.03
150 mg/kg	*M*	1.78 ± 0.38	8.92 ± 1.56	0.23 ± 0.06
	*F*	1.12 ± 0.43	9.28 ± 1.98	0.18 ± 0.06
300 mg/kg	*M*	1.58 ± 0.76	9.91 ± 3.39	0.20 ± 0.03
	*F*	2.53 ± 1.63	3.40 ± 2.06	0.38 ± 0.14
*P* value				
Solvent vs Saline	*M*	0.01^*^	0.82	0.01^*^
	*F*	0.16	0.55	0.1
75 mg/kg vs Saline	*M*	0.48	0.09	0.16
	*F*	0.00^**^	0.75	0.02^*^
150 mg/kg vs Saline	*M*	0.01^*^	0.94	0.01^*^
	*F*	0.01^*^	0.85	0.10
300 mg/kg vs Saline	*M*	0.18	0.63	0.00^**^
	*F*	0.02^*^	0.02^*^	0.01^*^

Results are presented as mean ± SD, *n* = 15/sex/group

*M* Male, *F* Female

Mean value indicates a significant difference (^*^P < 0.05, ^**^P < 0.01) compared to Saline

#### Coagulation function

The prothrombin times of female rats in solvent and the RODD 75 and 150 mg/kg groups were prolonged compared with those of rats in saline group on the 3rd day (15.1 ± 0.4 s, 14.9 ± 0.2 s, 14.9 ± 0.5 s vs 14.4 ± 0.1 s, *P* < 0.05). On the 14th day, the prothrombin time of female rats in RODD 300 mg/kg group (14.6 ± 0.3 s vs 12.1 ± 1.6 s, *P* < 0.05) were prolonged. However, these changes had no biologic significance.

#### Blood biochemistry indexes

Compared with saline group, the albumin and the albumin/globulin ratio of solvent and RODD groups decreased on the 3rd day (*P* < 0.05). Additionally, albumin decreased in solvent and RODD 75 and 150 mg/kg groups, alanine amino transferase increased in female rats in RODD 75 and 300 mg/kg groups and triglycerides increased in female rats in RODD 300 mg/kg group (*P* < 0.05) (Table 2).

**Table 2:** Effects of the subcutaneous injection of RODD on major blood biochemical indexes in rats

Group	Sex	Total protein (g/L)	Albumin (g/L)	Albumin, albumin /globulin ratio	Alanine amino transferase (U/L)	Triglyceride (mmol/L)
Saline	*M*	50.2 ± 3.1	30.4 ± 2.1	1.54 ± 0.09	38.8 ± 3.9	0.50 ± 0.10
	*F*	59.4 ± 4.2	35.4 ± 2.1	1.48 ± 0.08	24.6 ± 4.0	0.50 ± 0.07
Solvent	*M*	53.0 ± 2.5	28.6 ± 1.7	1.18 ± 0.08	36.6 ± 9.6	0.45 ± 0.11
	*F*	54.4 ± 1.0	30.4 ± 1.8	1.28 ± 0.08	31.8 ± 4.4	0.55 ± 0.16
RODD (mg/ kg)						
75	*M*	51.2 ± 1.3	28.0 ± 1.7	1.22 ± 0.15	37.6 ± 5.8	0.54 ± 0.15
	*F*	56.8 ± 0.8	31.6 ± 1.10	1.26 ± 0.09	29.8 ± 2.9	0.39 ± 0.10
150	*M*	54.0 ± 2.1	29.2 ± 0.4	1.20 ± 0.07	36.4 ± 4.6	0.44 ± 0.11
	*F*	58.6 ± 2.1	32.4 ± 1.1	1.24 ± 0.05	24.4 ± 4.3	0.59 ± 0.15
300	*M*	56.8 ± 1.7	29.3 ± 1.3	1.05 ± 0.06	44.5 ± 8.4	0.40 ± 0.06
	*F*	61.0 ± 9.5	33.7 ± 5.1	1.23 ± 0.06	44.0 ± 19.1	1.68 ± 1.06
*P* value						
Solvent vs Saline	*M*	0.16	0.17	0.00^**^	0.65	0.44
	*F*	0.04^*^	0.00^**^	0.01^*^	0.03^*^	0.56
75 mg/kg vs Saline	*M*	0.53	0.08	0.00^**^	0.71	0.66
	*F*	0.21	0.01^*^	0.00^**^	0.04^*^	0.10
150 mg/kg vs Saline	*M*	0.05	0.24	0.00^**^	0.40	0.37
	*F*	0.71	0.02^*^	0.00^**^	0.94	0.27
300 mg/kg vs Saline	*M*	0.01^**^	0.37	0.00^**^	0.21	0.13
	*F*	0.75	0.51	0.00^**^	0.04^*^	0.04^*^

Results are presented as mean ± SD, *n* = 15/sex/group

*M* Male, *F* Female

Mean value indicates a significant difference (^*^P < 0.05, ^**^P < 0.01) compared to Saline.

#### Observation after dissection

The rats were euthanized on the 3rd and 14th day after administration, rsepectively. On the 3rd day, the injection sites of rats were swollen with local dark redness or transparent pale yellow fluid exudation. On the 14th day, the injection sites presented local dark redness. All of these signs occurred in solvent and RODD groups.

#### Viscera weight and index

Compared with rats in saline group, liver weights of male rats in solvent group and RODD 75, 150 and 300 mg/kg groups increased on the 3rd day after administration (6.35 ± 0.34 g vs 6.87 ± 0.42 g, 7.21 ± 0.44 g, 7.84 ± 0.74 g, 7.52 ± 1.09 g, *P* < 0.05, *P* < 0.01). Females in RODD 300 mg/kg groups presented a similar results (6.38 ± 0.43 g vs 7.87 ± 1.44 g, *P* < 0.05). The thymic weight of male rats in solvent and RODD 300 mg/kg groups decreased (0.64 ± 0.07 g vs 0.39 ± 0.06 g, 0.34 ± 0.10 g, *P* < 0.05) and the thymic weights of female rats in solvent group, RODD 150 and 300 mg/kg groups were 0.36 ± 0.07 g, 0.41 ± 0.04 g and 0.37 ± 0.09 g, respectively (vs 0.58 ± 0.14 g, *P* < 0.05). The liver/body weight ratio of male rats in solvent and RODD groups significantly increased compared with those in saline group (3.80 ± 0.17, 3.77 ± 0.26, 3.94 ± 0.24 and 4.28 ± 0.21 vs 3.38 ± 0.12, *P* < 0.05) on the 3rd day. The thymus/body ratio of male rats in solvent and RODD 150 and 300 mg/kg groups decreased (0.22 ± 0.03, 0.28 ± 0.04, 0.19 ± 0.05 vs 0.34 ± 0.04, *P* < 0.05). Among all female rats, the spleen/body, liver/body and kidney/body weight ratios in RODD 300 mg/kg group increased (0.14 ± 0.05 vs 0.30 ± 0.04, 4.95 ± 0.56 vs 3.57 ± 0.07, 1.24 ± 0.19 vs 0.96 ± 0.08, *P* < 0.05). The ratio of thymus to body weight in solvent and RODD 300 mg/kg groups decreased (0.20 ± 0.04, 0.24 ± 0.08 vs 0.32 ± 0.09, *P* < 0.05). All indicators recovered on the 14th day.

#### Histopathological examination

**Fig. 5:**
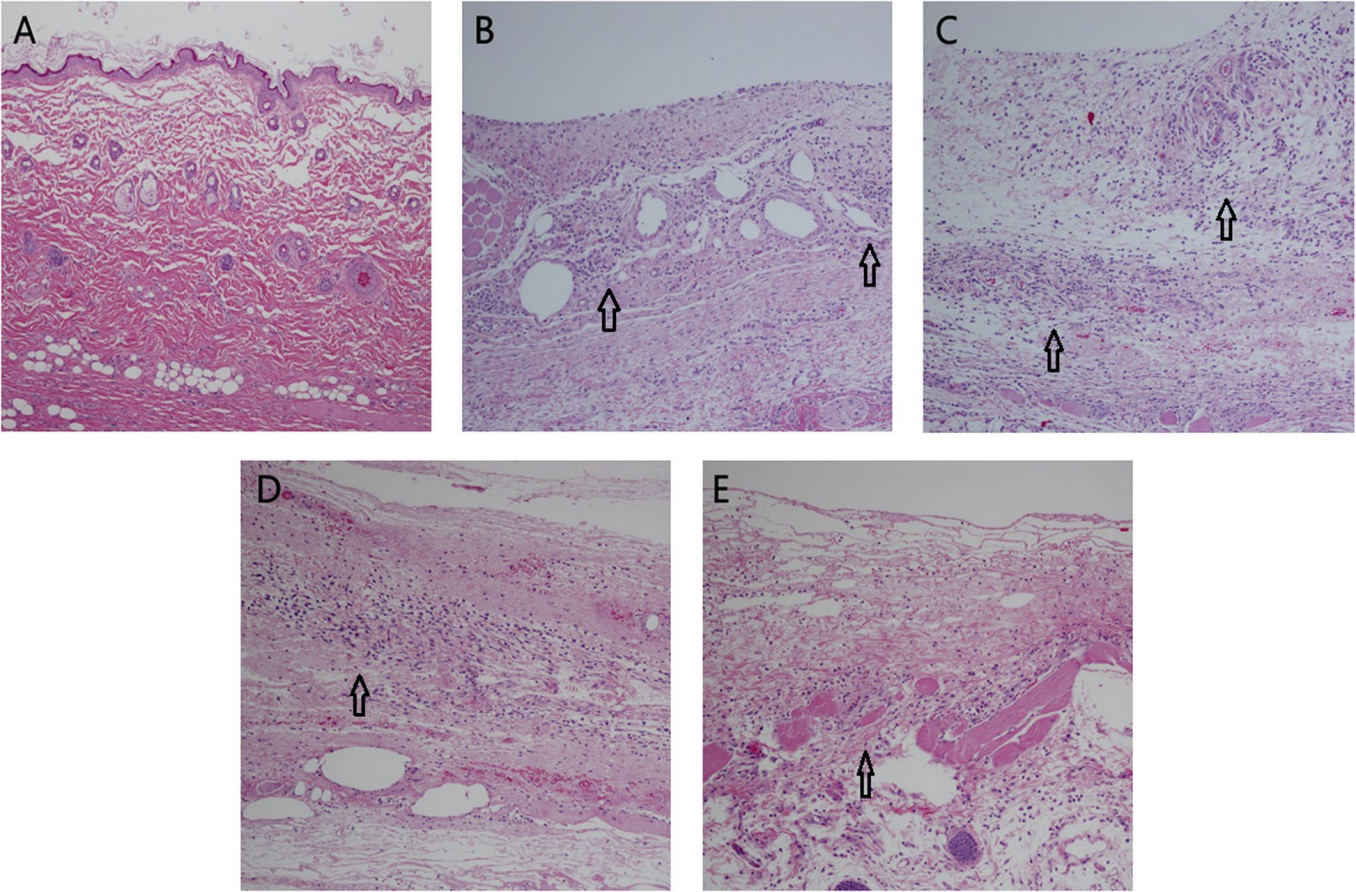
Histopathology of the injection site of RODD. **A**: saline group; **B**: solvent group; **C**: RODD 300 mg/kg group on the 3th day after administration. **D**: solvent group; **E**: RODD 300 mg/kg group on the 14th day after administration, showed a recovery trend, but still had inflammatory cell infiltration, granulation tissue formation/fibrosis. The arrows showed inflammatory cell infiltration. Bar = 100 μm

**Fig. 6:**
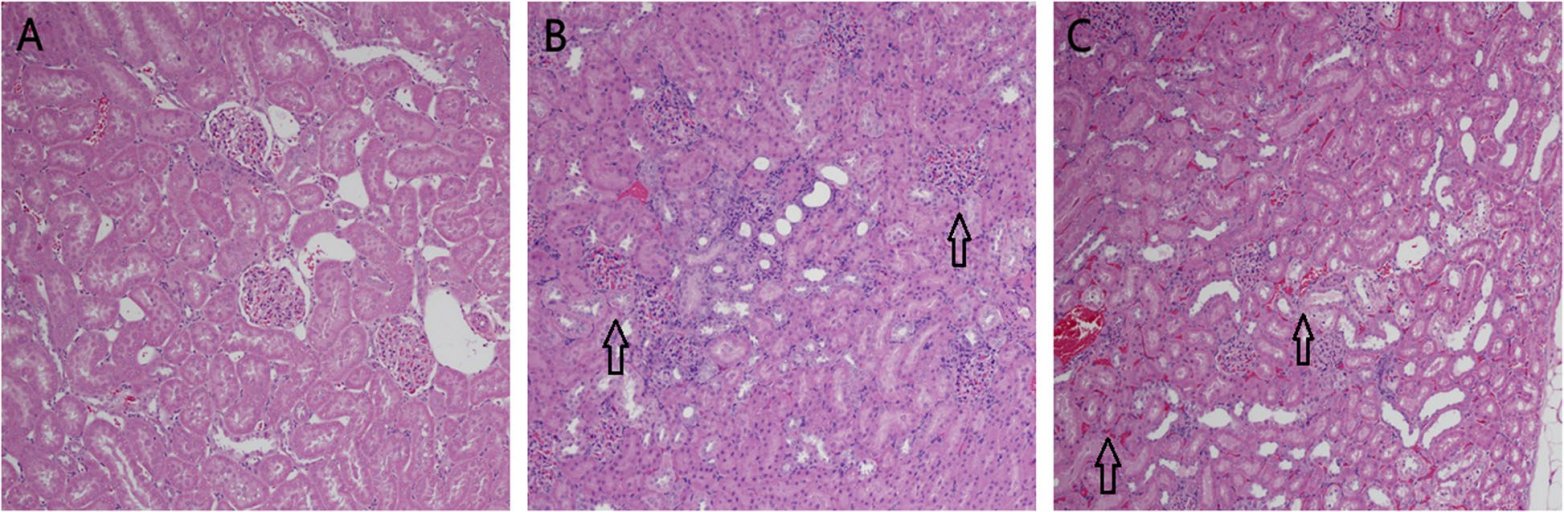
Renal histopathology after administration of RODD. On the 3th day after administration, solvent group (**B**) and RODD 300 mg/kg group (**C**) compared with saline group (**A**), interstitial listed nuclear cell infiltration and, vacuoles increased significantly. The arrows showed occluded capillaries. Bar = 50 μm

As shown in Fig. 5, on the 3rd day after administration, microscopic examination revealed mild to moderate subcutaneous inflammation with large areas of necrosis or inflammatory cell infiltration in solvent and RODD groups. In RODD 300 mg/kg group, two males and two females presented mild lobular central hepatocyte hypertrophy corresponding to the liver weight increase. Basophilic renal tubules, inflammatory cell infiltration and some capillaries occluded, occurred in 3 female rats in RODD 300 mg/kg group (Fig. 6), and thymus and/or spleen lymphocytes were atrophied in solvent and RODD 300 mg/kg group and organ weight decreased. On the 14th day after injection, the rats of solvent and RODD 75, 150, 300 mg/kg groups suffered mild chronic inflammation, granulation tissue or fibrosis, indicating a tissue repair process. Most of the histopathological abnormalities in rats disappeared, including lobular central hepatocyte hypertrophy, mild basophilic renal tubules and lymphocytic atrophy in thymus, etc.

### Toxicokinetics

#### Methods validation

There was no any interference peak near the peaks of ropivacaine and inner standard, suggesting that the analysis method was specific. Within the range of 4⁓1000 ng/mL, the curve equation was Y = 6.06X + 9.46 and the correlation coefficient met the requirements (*r *= 0.9996). The accuracy was 85.9%⁓105.5% and the limit of quantitation was 4 ng/mL. The accuracy of the ropivacaine quality control sample was 85.2%⁓114.7% and the intrabatch and interbatch precisions were 0.89%⁓3.16% and 3.48%⁓9.86%, respectively. The recovery rates were 109.2% ± 5.5%, 100.7% ± 1.7% and 100.5% ± 0.8% at the concentrations of 12, 80 and 800 ng/mL. The matrix effects were 94.2% ± 2.6%, 94.0% ± 0.8% and 96.1% ± 0.7%, respectively. The recovery of plasma samples in stability study were 100.9%⁓102.2%, 100.0% ~ 104.7%, 95.2%⁓102.8% and 102.5%⁓107.0% immediately after preparation, after 4 h at room temperature, after 7 days at -15 °C, after 4 weeks at -15 °C, indicating that the samples had good stability.

**Fig. 7:**
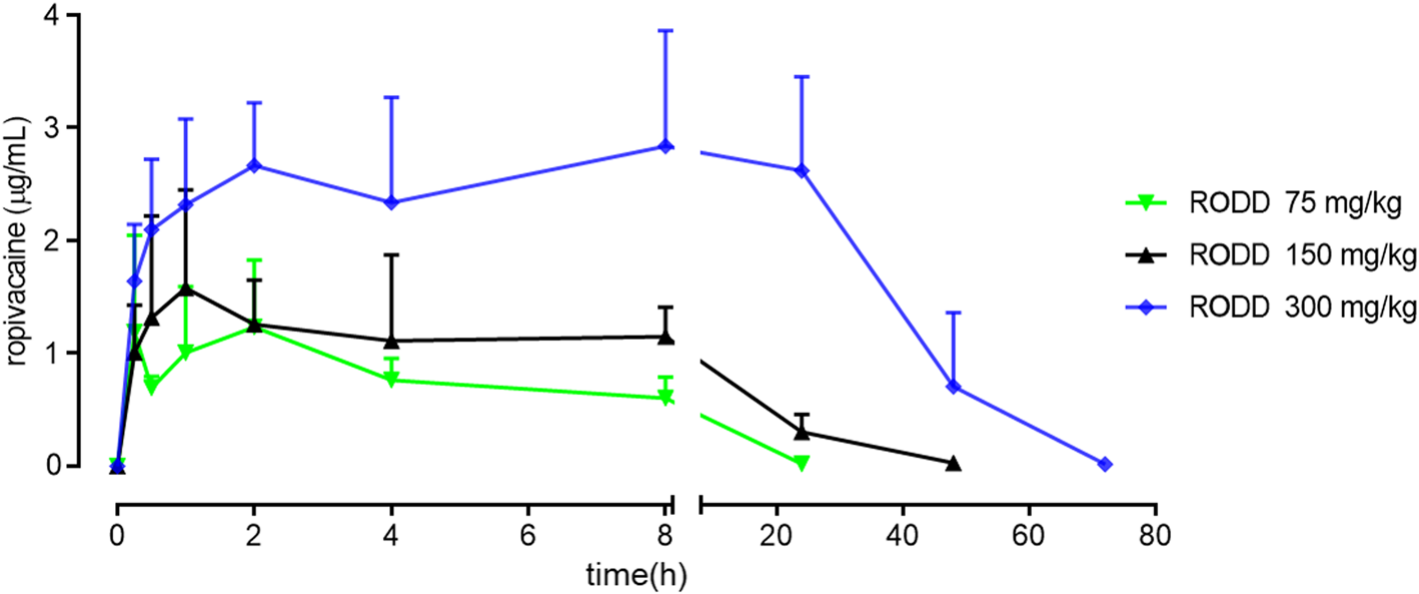
Plasma concentration–time curve after the subcutaneous injection of RODD in rats. *n* = 18 (half male and half female). The subcutaneous injection of the RODD in rats had a stable release without sudden release

Figure 7 showed concentration–time curve of RODD. The area under the curves were positive to doses of ropivacaine. The resease of ropivacaine was stable. The peak time of release was about 1 ~ 2 h, and lasted 24 h. Table 3 showed the toxicokinetic parameters of ropivacaine in rats. The t_1/2_ of ropivacaine were from 3.60 to 7.01 h in dose 75⁓300 mg/kg. The C_max_ and AUC_(0–24 h)_ of ropivacaine were positively correlated with the doses. When the dose ratio was 1:2:4, the C_max_ ratio of ropivacaine was 1:1.27:2.29 and the AUC_(0–24 h)_ ratio was 1:2.07:9.25. The C_max_ ratio of ropivacaine of female and male rats ranged from 0.51 to 0.95 and the AUC_(0–24 h)_ ratio ranged from 0.82 to 0.97 among the RODDs (75⁓300 mg/kg). The exposure of ropivacaine was slightly higher in male rats than in females.

**Table 3:** Toxicokinetic parameters of ropivacaine in the plasma of rats

Dose(mg/kg)	t_1/2_(h)	T_max_(h)	C_max_(μg/mL)	AUC_(0–24 h)_ (h·µg/mL)	AUC_inf_(h·µg/mL)
75	3.60 ± 0.68	1.21 ± 0.68	1.24 ± 0.59	11.65 ± 1.58	11.77 ± 1.64
150	7.87 ± 1.58	0.71 ± 0.33	1.58 ± 0.87	24.14 ± 3.48	25.34 ± 4.88
300	7.01 ± 1.20	8.00 ± 8.29	2.84 ± 1.02	107.80 ± 12.98	148.18 ± 88.19

Results are presented as mean ± SD, *n* = 18.

## Discussion

In oil delivery depot (ODD), organic solvent is mainly used as solvent, and vegetable oil or phospholipid is used as dispersants. The ODD has high drug-loading capacity. The depot is usually injected intramuscularly or subcutaneously, and can release slowly in the injection site. ODD is commonly used to diseases of the reproductive or psychiatric systems and the drugs include haloperidol decanoate, flupentixol decanoate, nandrolone phenylpropionate, testosterone enanthate, etc. [13, 14]. A phospholipid-based oily gel formulation of ropivacaine freebase suspension was designed, which extended the nerve block time in guinea pigs by three times and presented good biocompatibility [15]. In this study, RODD was expected to achieve longer analgesic effect.

In pharmacodynamics, the sciatic nerve of rabbits were rapidly blocked with RAI 0.9 mg, and the block quickly disappeared in about 2 h. The results suggested that release of ropivacaine was quick. RODD 0.9 mg did not significantly block nerve conduction, suggesting that the release of ropivacaine was too slow to reach an accumulate concentration for nerve blocking. RODD 3 mg blocked nerve as fast as RAI, and the action duration was about 8 times that of RAI, suggesting that RODD had obvious sustained release effect. After RODD 3 mg, nerve conduction velocity could recover, demonstrating that the blocking is reversible.

In toxicology, available data indicated that the maximum volume in rats was 10 mL/kg [16] and the concentration of RODD was 30 mg/mL. Thus, the maximum dose for ropivacaine toxicology studies was 300 mg/kg. In addition, we simulated injection around the surgical incision and conducted multipoint subcutaneous delivery in rats [17]. In RODD 300 mg/kg group, some rats died and experienced local swelling, acute local inflammation, scattered dilatation of renal tubules, mononuclear infiltration of liver, diffuse atrophy and central system abnormalities. Toxicological study showed that the toxic target organs were mainly in the liver, kidney and central system, and RODD had irritation. Rats in RODD 150 and 75 mg/kg groups were survived, there was no significant toxicity in dose of RODD 75 mg/kg. In pharmacodynamics, the dose of RODD 3 mg/rabbit was 1.2 mg/kg body weight, which was much lower than non-toxic dose of RODD 75 mg/kg. In clinic, the dose of subcutaneous infiltration ropivacaine was about 3 mg/kg [18]. Therefore, RODD is safe.

The toxicokinetics showed the release of ropivacaine was slow and stable. The peak time of release was about 1⁓2 h, and lasted 24 h. There was no a sudden release. Those suggest that RODD release is slow and stable. The rats in RODD 75 mg/kg did not showed any toxicity, and its peak concentration 1.24 ± 0.59 µg/mL should be nontoxic concentration. The rats in RODD 150 or 300 mg/kg showed abnormal behaviors or partial death, and its C_max_ 1.58 ± 0.87 or 2.84 ± 1.02 µg/mL should be poison to lethal concentration, which is closer to concentration of patients with neurotoxicity of 2.7 ± 0.46 µg/mL in clinic [19].

## Conclusions

RODD releases ropivacaine slowly, and show a stable and longer analgesic effect with a large safety range.

## Data Availability

The data sets used and/or analysed in the current study are available from the corresponding author on reasonable request.

## Abbreviations

AUC: Area under the curve
C_max_: Maximum concentration
LC–MS/MS: Liquid chromatography tandem mass spectrometry
ODD: Oil delivery depot
RAI: Ropivacaine aqueous injection
RODD: Ropivacaine oil delivery depot
t_1/2_: Half-life period
